# Study of the Solid-State Synthesis of Nickel Ferrite (NiFe_2_O_4_) by X-ray Diffraction (XRD), Scanning Electron Microscopy (SEM) and Raman Spectroscopy

**DOI:** 10.3390/ma14102557

**Published:** 2021-05-14

**Authors:** Chloé Cherpin, Derek Lister, Frédéric Dacquait, Lihui Liu

**Affiliations:** 1 Commissariat à L’énergie Atomique et aux Énergies Alternatives, Institut de Recherche sur les Systèmes Nucléaires pour la Production D’énergie Bas Carbone, Département de Technologie Nucléaire Cadarache, F-13108 Saint-Paul-Lez-Durance, France; frederic.dacquait@cea.fr; 2 Chemical Engineering Department, University of New Brunswick, P.O. Box 4400, Fredericton, NB E3B 5A3, Canada; dlister@unb.ca (D.L.); lihui@unb.ca (L.L.)

**Keywords:** nickel ferrite, solid-state reaction, X-ray diffraction, Raman spectroscopy, scanning electron microscopy

## Abstract

Spinel ferrite compounds continue to receive a lot of attention due to their unique properties. Among the numerous synthesis routes existing, the solid-state method was applied for the production of nickel ferrite, by introducing the use of a quartz vial. A mixture of nickel oxide (NiO) and hematite (Fe_2_O_3_) was ground and vacuum-sealed in the vial and different thermal treatment programs were tested. The resulting particles were characterized by X-ray diffraction (XRD), scanning electron microscopy (SEM) and Raman spectroscopy. For temperatures, below 1000 °C, the solid-state reaction is not complete as nickel oxide (NiO) and hematite (Fe_2_O_3_) are still present. The reaction time is a decisive parameter for the morphology of the particles obtained. If, for different reaction times, the particle size distribution is always between 0.3 and 1.7 µm, a longer reaction time leads to the formation of dense, interconnected clusters of particles. Optimal parameters to synthesize a pure phase of spherical nickel ferrite were sought and found to be a reaction temperature of 1000 °C for 72 h.

## 1. Introduction

Spinel ferrites have received a lot of attention since their discovery due to their interesting properties and commercial potential. While ferrites refer to ceramics of the general formula AFe_2_O_4_ (where A is a divalent cation) that for some applications may be considered as mixed oxides containing hematite (Fe_2_O_3_), spinels are actually cubic crystalline structures based on the mineral spinel MgAl_2_O_4_ [1]. The so-called normal spinels have the divalent cation (Mg^2+^ in the last example) in tetrahedral sites in the crystal lattice and the trivalent cations (Al^3+^ in the example) in octahedral sites. In inverse spinels, the divalent cations are in octahedral sites and the trivalent cations are distributed between tetrahedral and octahedral sites. All the ferrites are inverse spinels. Because of their excellent magnetic and electrical properties, associated to their thermodynamic stability and high corrosion resistance, their fields of application are abundant.

Nickel ferrite (NiFe_2_O_4_) is used in various applications such as information systems and data storage devices [2,3], sensors [4], water purification systems [5] etc. It is also a major corrosion product that creates fouling and contamination problems in the high-temperature water circuits of power plants [6,7,8,9,10]. Its synthesis is of wide-ranging interest.

A 2020 publication [11] has reviewed the numerous routes to synthesize nickel ferrite. The most used routes consist of co-precipitation [12,13], sol-gel [14,15,16,17], solid-state [18,19,20,21,22,23,24,25] and micro-emulsion method [26]. In this study, a solid-state method is developed to synthesize uniform particles of nickel ferrite for research into its properties as a colloid. The method is of particular interest for its simplicity and high reproducibility, given that no by-products are formed and that the stoichiometry is easily controlled.

A solid-state reaction route that does not involve milling was developed. Thus, the different reagents as powders are mixed and ground together in a mortar and pestle before undergoing a thermal treatment, which ultimately leads to the full conversion into the desired ferrite. This method has been applied to the synthesis of several ferrites, including magnetite (Fe_3_O_4_) and cobalt ferrite (CoFe_2_O_4_), and is equally useful for other spinels. The method is very flexible, as it allows the desired compound to be tailored in advance so that even non-stoichiometric compositions can be readily synthesized.

The basic method was used by Deydier [27] to form magnetite. That study showed that the solid-state reaction forming magnetite occurs at temperatures above 600 °C. Above this threshold, the temperature influences the morphology of the product particles, as the increased sintering distorts the particle shape from the desired sphericity. The reaction time for the synthesis of magnetite also governs the morphology of the powder, as it was found that the mean particle size increases with the reaction time. In addition, this study showed that an increase in the heating rate leads to the formation of dense, interconnected clusters. It is therefore suggested that a low heating rate be used to form colloidal particles.

In the present report, the influence of reaction time and temperature on the morphology and purity of nickel ferrite synthesized by the solid-state reaction method was investigated. SEM, XRD and Raman spectrometry were used to characterize the obtained powders and the optimal parameters were determined.

## 2. Materials and Methods

### 2.1. Sample Preparation

The formation of NiFe_2_O_4_ ferrite by the solid-state method relies on the following reaction:

NiO + Fe_2_O_3_ → NiFe_2_O_4_

**Figure 1:**
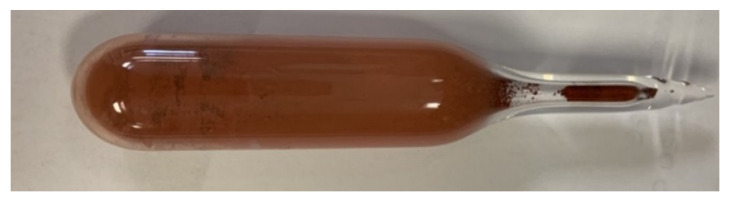
Quartz glassware used for the synthesis.

Stoichiometric amounts of nickel oxide (NiO) and hematite (Fe_2_O_3_) were used as starting reactants. Nickel oxide (99% purity) was purchased from Fisher Scientific(Ottawa, ON, Canada) and hematite (96% purity) from Sigma-Aldrich(Oakville, ON, Canada). In order to produce a homogeneous mixture for a reaction sample, a mass of 3.2 g of nickel oxide and 7.1 g of hematite were mixed and thoroughly ground with a pestle in a mortar with the addition of a few drops of ethanol. This grinding was continued until the ethanol had evaporated and the process was repeated four times. The powder was then rapidly vacuum-sealed in a quartz vial around 12 cm long (see Figure 1).

The vacuum sealing is essential to ensure that no oxidation occurs during the heating. The vial containing the mixture was placed inside a furnace exposed to surrounding air. The appropriate heating cycle was set up as described in the test matrix below (see Table 1). Once the furnace had cooled to room temperature at the end of the cycle, the vial was removed and carefully broken at the tip to avoid any quartz fragments from contaminating the powder. The powder was once again crushed/ground in a mortar and pestle and finally characterized.

Deydier [27] showed that the sintering and formation of dense, interconnected clusters results from a high heating rate. Therefore, for the formation of homogenous spherical colloids, a low heating rate of 0.5 °C/min was fixed for all the experiments. The following test matrix was established (Table 1):

### 2.2. Characterization

X-ray diffraction patterns of samples were recorded with a spectrometer (Bruker AXS D8 Advance, Bruker, Milton, ON, Canada). The fine powder samples were packed into the circular well on the sample-holder, after which it was placed on the sample stage for scanning. The X-ray source is a sealed 2.2 kW Cu tube, operated with a voltage of 40 kV and current of 25 mA, and the detector is a Peltier-cooled solid-state Si(Li) device with a useful energy range of 1 to 60 KeV. Samples were scanned in the 2θ range of 5–80° with a step size of 0.02° and a step time of 1.0 s. No correction was made for Kβ radiation. The raw data obtained from the spectrometer was analyzed and refined by the program EVA (Bruker, Milton, ON, Canada).and some also analyzed with X’pert Highscore (version 4.9, Malvern Panalytical, Montréal, QC, Canada) to determine the amounts of constituents. An average of five determinations was recorded for each the five samples analyzed.

Raman spectrometrywas performed using a Renishaw inVia Raman microscope (Renishaw, Mississauga, ON, Canada) incorporating a He-Ne laser with a wavelength of 633 nm and a power of 17 mW. The exposure time was 100 s. For each sample, an average of five measurement points was taken to confirm the homogeneity of the powder. Out of the five spectra obtained, a representative spectrum was chosen and analyzed for each sample.

Scanning electron microscopy (SEM) (JEOL 6400, Jeol Canada, Inc., St-Hubert, QC, Canada) was conducted to obtain further information about the particle size and morphology. The samples were not further ground and simply packed in a powder sample holder. The working distance was kept around 14 mm and the pictures were obtained with secondary electron imaging with an accelerating voltage of 15 kV. Analysis was performed at the UNB Microscopy and Microanalysis.

## 3. Results and Discussion

### 3.1. Effect of Temperature

**Figure 2:**
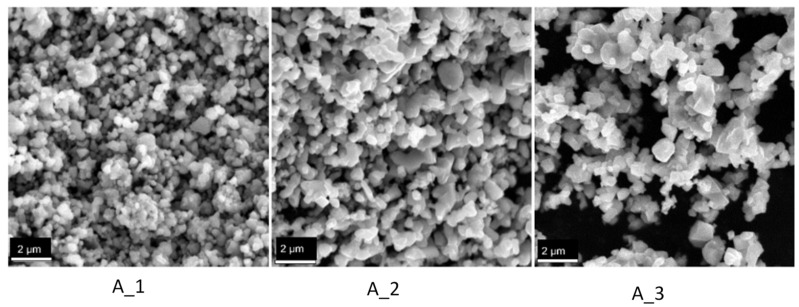
SEM images obtained for the samples A_1 (600 °C for 48 h), A_2 (800 °C for 48 h) and A_3 (1000 °C for 48 h).

The SEM images obtained for the samples A_1, A_2 and A_3 are shown in Figure 2.

Figure 2 indicates that a higher temperature forms bigger particles and clusters of particles from sintering.

**Figure 3:**
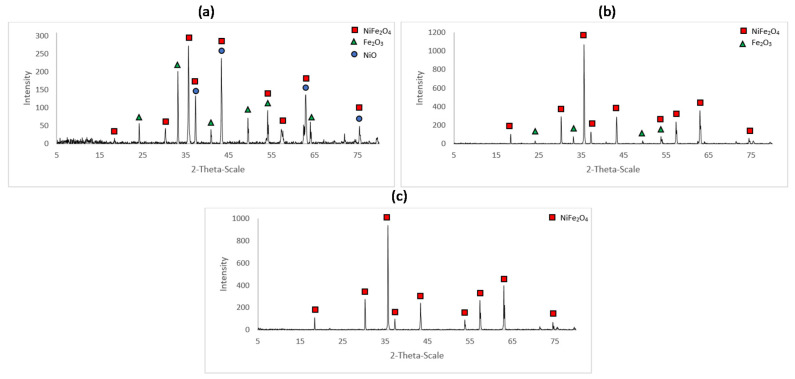
XRD spectrum for the (**a**) sample A_1 (600 °C for 48 h), (**b**) sample A_2 (800 °C for 48 h) and (**c**) sample A_3 (1000 °C for 48 h).

The XRD patterns for the samples A_1, A_2 and A_3 are shown in Figure 3. The characteristic peaks for NiO, Fe_2_O_3_ and NiFe_2_O_4_ were identified using the respective ICDD (International Center for Diffraction Data) cards 44-1159, 33-0664 and 54-0964.

Figure 3 indicates that the samples A_1 and A_2 were composed of more than one constituent. Data were processed with X’pert Highscore software. This software quantifies the amount of a phase in a mixture using the Rietveld method. This well-used method [28] consists in fitting an experimental spectrum to a calculated pattern based on the hypothetical crystal features. This allows to determine the relative amount of each phase identified within a given sample. The percentages of NiO, Fe_2_O_3_ and NiFe_2_O_4_ were determined for the samples A_1 and A_2 (Table 2).

For the analyzed portions of sample A_2, the results indicate that there had been a surplus of Fe_2_O_3_ or a dearth of NiO in the starting mixture. Clearly, the mixture was not homogeneous. Similarly, for sample A_1, where the reaction was far from complete, the ratio of unreacted Fe_2_O_3_ to NiO indicates that there was a stoichiometric surplus of nickel oxide. Figure 3 indicates complete conversion to NiFe_2_O_4_.

From the X-ray diffraction spectra obtained for the samples A_1, A_2 and A_3, it is possible to follow the evolution of the solid-state reaction. The conversion of nickel oxide and hematite to nickel ferrite is not completed for a temperature of 600 °C for 48 h. Indeed, as shown in Table 2, characteristic peaks of NiO and Fe_2_O_3_ in non-stoichiometric proportions are obtained in sample A_1. For sample A_2 at 800 °C, due to the surplus of hematite or dearth of nickel oxide coming from the incomplete mixing, the reaction is completed but surplus hematite remains. As confirmed by Figure 3 for sample A_3, for which the constituents were thoroughly mixed, the reaction is certainly completed at 1000 °C.

**Figure 4:**
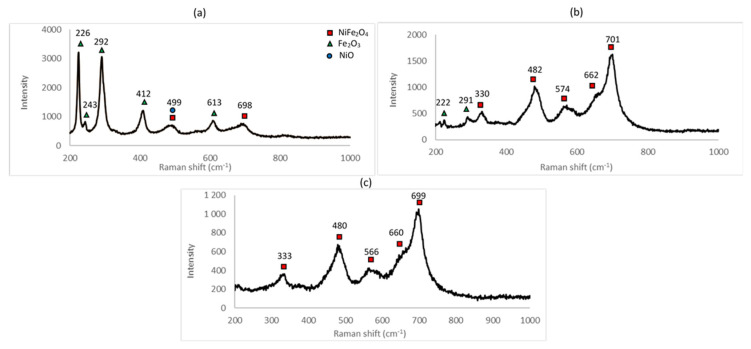
Raman spectrum of (**a**) sample A_1 (600 °C for 48 h), (**b**) sample A_2 (800 °C for 48 h) and (**c**) sample A_3 (1000 °C for 48 h).

Raman spectra for the samples A_1, A_2 and A_3 are shown in Figure 4.

**Figure 5:**
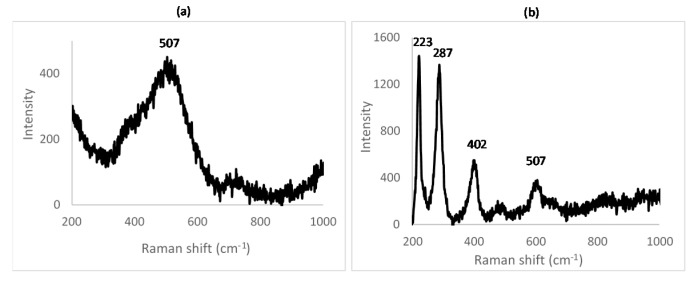
Raman spectrum obtained for (**a**) NiO and (**b**) Fe_2_O_3_.

In order to identify the Raman-shift peaks for unreacted nickel oxide and hematite, Raman spectroscopy was conducted on the NiO and Fe_2_O_3_ powders used as the starting reagents (see Figure 5).

From the spectra, it is clear that NiO is hard to identify, having a broad, weak signal around 500 cm^−1^. Its peak will be swamped by nearby, stronger peaks from other species. Hematite, however, has a strong signal with sharp peaks around 230 and 290 cm^−1^ accompanied by smaller peaks around 400 and 610 cm^−1^. This is confirmed by the study of Shebanova [29].

The identification of nickel ferrite is confirmed by the Guzonas study [30], where nickel ferrite shows peaks around 330, 485 and 700 cm^−1^, accompanied by a shoulder around 660 cm^−1^.

From the Raman spectra obtained for the samples A_1, A_2 and A_3, the different peaks were identified. All the Raman spectra show peaks attributable to NiFe_2_O_4_. The peaks characteristic of hematite are clearly present for the samples A_1 and A_2, thus confirming the results obtained by XRD. No trace of hematite is seen in sample A_3, confirming that the reaction was completed at 1000 °C.

The same conclusion as that obtained from the XRD spectra can be deduced: for a furnace temperature of 600 °C, the peaks from two unreacted components, NiO and Fe_2_O_3_, indicate that the reaction was incomplete at least within the reaction time of 48 h. As described previously, at 800 °C, the reaction was probably complete with surplus Fe_2_O_3_ indicated (see Table 2). This surplus can either come from incomplete mixing or from the precision pertaining to the weighing of the initial reagents. At 1000 °C, the reaction was complete.

### 3.2. Effect of the Duration of the Thermal Treatment

**Figure 6:**
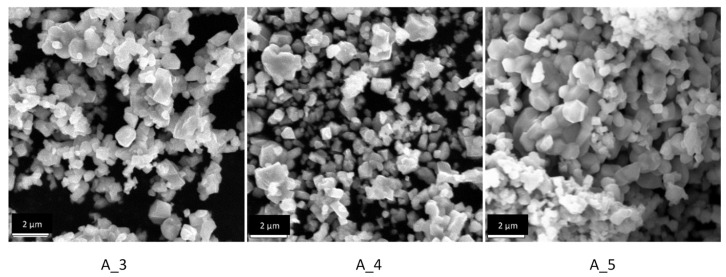
SEM images for the samples A_3 (1000 °C for 48 h), A_4 (1000 °C for 72 h) and A_5 (1000 °C for 96 h).

The SEM images obtained for the samples A_3, A_4 and A_5 are shown in Figure 6.

As the reaction time increases at 1000 °C, the particles tend to become more faceted and form interconnected clusters. The average particle size also slightly increases with the reaction time but still stays within the range of 0.3 to 1.7 µm.

**Figure 7:**
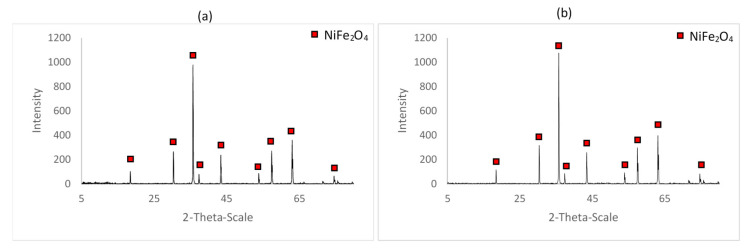
XRD spectra for samples (**a**) A_4 (72 h at 1000 °C) and (**b**) A_5 (96 h at 1000 °C).

The XRD spectra obtained for the samples A_4 and A_5 were very similar to that for A_3, as shown on Figure 7. They show peaks characteristic of nickel ferrite, with no trace of hematite nor nickel oxide.

**Figure 8:**
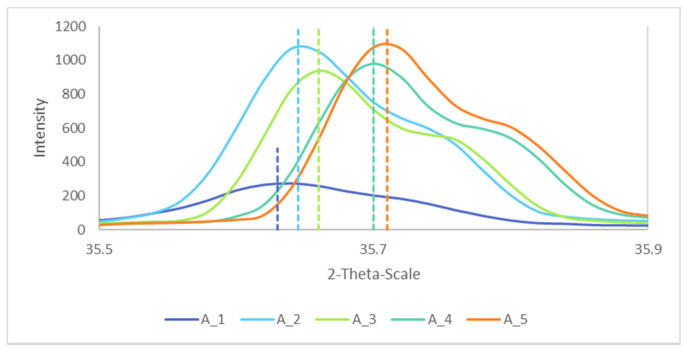
Bragg peaks of A_1, A_2, A_3, A_4 and A_5 for h = 3, k = 1 and l = 1, with their respective maxima as the dotted lines.

Based on their XRD spectra, the lattice parameters of the samples were calculated. Figure 8 shows the Bragg peaks for the different samples. The largest diffraction angles were for the higher temperature of reaction, and corresponded to lower values of the lattice parameter, confirmed elsewhere [31].

The calculated lattice parameter was found to be in good agreement with the reported value by Shafer [32] of 0.8339 nm (see Table 3).

**Figure 9:**
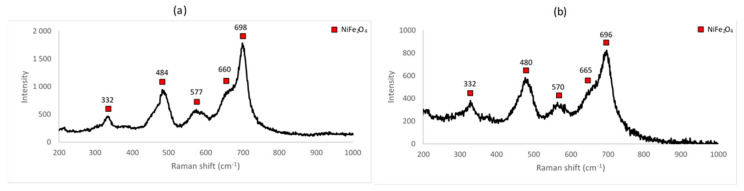
Raman spectrum for (**a**) sample A_4 (1000 °C for 72 h) and (**b**) A_5 (1000 °C for 96 h).

Raman spectra for the samples A_4 and A_5 are shown in Figure 9 below. They are characteristic of nickel ferrite, showing peaks that are identifiable in the spectra of all the samples. Differences among all of them are attributed to the presence of the unreacted constituents and/or measurement artefacts (variations in the power of the laser between measurements, properties intrinsic to the samples, etc.).

The analyses indicate that a temperature of 800–1000 °C and a reaction time of 72 h should be optimum conditions to form pure colloidal nickel ferrite with a close-to-spherical shape and narrow size range.

## 4. Conclusions

NiFe_2_O_4_ was prepared by a solid-state method and characterized with XRD, SEM and Raman spectroscopy. The use of a quartz vial in which the stoichiometric mixture of nickel oxide and hematite is vacuum sealed is a convenient innovation, as it requires no specific gas in the furnace. It is noted that the method produces no oxide such as magnetite, Fe_3_O_4_, that might otherwise be produced by the concomitant reduction of hematite. The reactant oxides must be thoroughly mixed to avoid regions of unreacted components in the finished product.

The influences of reaction time and temperature on the composition and morphology of the particles were studied. XRD and Raman spectroscopy revealed that the solid-state reaction leads to a full conversion of NiO and Fe_2_O_3_ into NiFe_2_O_4_ for a temperature of 800–1000 °C. At 600 °C, the reaction was incomplete after 48 h. SEM has shown that a longer reaction time than 72 h, namely 96 h, leads to the formation of interconnected and dense clusters of particles and to the deformation of initially spherical particles, which develop facets with continuing exposure.

The optimal parameters for the formation of colloidal nickel ferrite have thus been determined and confirmed with XRD and Raman spectra. A temperature of 800–1000 °C is recommended to form a pure phase of nickel ferrite. The duration of the thermal treatment can be adapted according to the desired size and agglomeration of particles, but 72 h is the recommended choice to form almost spherical and uniform particles.

## Figures and Tables

**Table 1:** Test matrix with the thermal treatment conditions used for each sample.

Sample	Reaction Time	Furnace Temperature
A_1	48 h	600 °C
A_2	48 h	800 °C
A_3	48 h	1000 °C
A_4	72 h	1000 °C
A_5	96 h	1000 °C

**Table 2:** Composition of the samples A_1 (600 °C for 48 h) and A_2 (800 °C for 48 h).

Sample	NiO	Fe_2_O_3_	NiFe_2_O_4_
A_1	26%	36%	38%
A_2	-	9%	91%

**Table 3:** Lattice parameter of the samples A_1 (600 °C for 48 h), A_2 (800 °C for 48 h), A_3 (1000 °C for 48 h), A_4 (1000 °C for 72 h) and A_5 (1000 °C for 96 h).

Sample	Lattice Parameter (a = b = c)
A_1	a = 0.8350
A_2	a = 0.8346
A_3	a = 0.8343
A_4	a = 0.8334
A_5	a = 0.8332

## Data Availability

The data presented in this study are available on request from the corresponding author.
